# Supplementary material for: Patterns of Intron Gain and Loss in Fungi
Source: PLoS Biol. 2004 Nov 30;2(12):e422. doi: 10.1371/journal.pbio.0020422 (PMC532390; doi:10.1371/journal.pbio.0020422)
Supplement: Table S1 — Also available at http://genes.mit.edu/NielsenEtAl/. (4.3 MB ZIP). [file pbio.0020422.st001.zip › NielsenEtAl/html/872.html]

AN0922.1.NCU00493.1.MG05676.1.FG05436.1


```
 CLUSTAL W (1.82) Multiple Sequence Alignments - Introns Inserted


Sequence 1: MG05676.1	514 aa
Sequence 2: FG05436.1	527 aa
Sequence 3: NCU00493.1	512 aa
Sequence 4: AN0922.1	516 aa
Alignment Length: 533 aa
Number Identitical Residues: 305 aa
Alignment Score (without introns) 13795


MG05676.1 	M0VVLAASICTRGGKAVLSRQFREMPRSRIEALLASFPKLADSGTQHTTVEQDNVRFVYQ
NCU00493.1	M0VVLAASICTRGGKAVLARAFNDIKRSRIEALLASFPKAADSGTQHTIVEQDNVRFVYQ
FG05436.1 	M0VVLAASICTRGGKAVLSRQFREMPRSRIEALLASFPKLADSGTQHTIVEQDNVRFVYQ
AN0922.1  	M0VVLAASICTRGGKAVLSRQFREIARSRVEALLASFPKLADSGTQHTTVEQDNVRFVYQ
          	* ****************:* *.:: ***:********* ******** ***********

MG05676.1 	PLDELYMVLITNRQSNILQDIDSLHLFAQVVTSTCKSLDEREILKNAYELLSAFDELVTL
NCU00493.1	PLDELYMVLITNKQSNILQDIDSLHLFAQVVTSTCKSLDEREILRNAYELLSAFDELVTL
FG05436.1 	PLDELYMVLITNRQSNILQDIDSLHLFAQVVSSTCKSLDEREIVKNAYELLSAFDELVTL
AN0922.1  	PLDELYIVLITNRQSNILQDIDSLHLFAQVTTSICKSLDEREILRNAFELLSAYDELVTL
          	******:*****:*****************.:* *********::**:*****:******

MG05676.1 	GYRENLTISQIKTFLEMESHEERIQEIIAR0NKELEATEERKRKAKQLEMQRKESARSGR
NCU00493.1	GYRENLTISQIKTFLEMESHEERIQEIIAR~NKELEATEERKRKAKQLEMQRKESARTGR
FG05436.1 	GYRENLTISQIKTFLDMESHEERIQEIIAR0NKELEATEERKRKAKQLEMQRKDAARSGR
AN0922.1  	GYRENLSLSQIKTFLEMESHEERIQEIIER0NKELEASEERKRKAKQLEMQRKEAARNSR
          	******::*******:************ * ******:***************::**..*

MG05676.1 	P---------------GGAQRAPVYPTYTPPSRPAVTDTYDTYEAEKNKS--K2FTAPKG
NCU00493.1	S----------------AVPRTPVYPTYTPPVRPAVTESYDTYEAEKNKV--K2FNAPKG
FG05436.1 	PAAGAMGGMGGGMGSSGGMPRSPSYPSYNPPSQ-SNTSTYDSYEAEKNKSFGK2PLAPKA
AN0922.1  	N----------------IAPRAPSYPVYTPPSRPAVPDTYDSYEAEKKKTFAK2PLPTRG
          	                    *:* ** *.** :.: ..:**:*****:*  .*   ..:.

MG05676.1 	KGMQLGKKSKTTDMFERVRGDMGNEIDDT-PLVAPTASAAPLASEPPRRQSVGTDRDSIH
NCU00493.1	KGMQLGKKSKNTDIFERVKGDLGHEAEEA-PLVPVAAPAAAAEPAAS-RVSSTLDRDAIH
FG05436.1 	KGMQLGKKSKTTDMFERVRGDMGSEIDHS-PLVTPAPQQQAETPEP--RVSSTLDRDAIH
AN0922.1  	KGMQLGKKSKTTDIYEKVRGDLGPEAEESSPLVTPQASTPVAERVSSARPSLSADRDPIH
          	**********.**::*:*:**:* * :.::***.  .        .. * *   ***.**

MG05676.1 	ITVSENITAKISREGTLNSLGVKGDLNLRVSDPTMTKIKLQLVANPTHGAQFRTHPNVDR
NCU00493.1	VLINEAISAKLSRDGALESLSVSGDLQLRVSDPSLSRIKLNLSATPTHGAQFRTHPNVDK
FG05436.1 	IAISETISAKLSREGAVNSLAVSGDLVLRISDPSLTKIKLGLQAVASHGVQFRTHPNVDR
AN0922.1  	ITIAETISASLTRDGALKSFEVKGDLQLRISDPAFTKLRLDLLANPTHGAQFRTHPNVDK
          	: : * *:*.::*:*:::*: *.*** **:***::::::* * * .:**.*********:

MG05676.1 	NLFNSSKVIQMSKADRGFPVNNSVGVLRWMAT-PKADDTSALPISFTVWVNKGSDGNCTL
NCU00493.1	NVFNSSQAIQLSQITKGFPLNNSVGVLRWRAS-PRVDDTSALPISFTVWVNKGSDGNSTL
FG05436.1 	NLFNSSKVIQMSNTARGFPVNNAVGVLRWRAS-PKVDDPSACPITFTVWINKDAD-KYNI
AN0922.1  	AAFSNSSIIQLKDTSKRFPANNSIGVLRWRVAGSGSDNADVLPITFTVWLNKGSD-STTV
          	  *..*. **:..  : ** **::***** .:..  *:... **:****:**.:* . .:

MG05676.1 	TVEYELTGGDELRDVSVSIPYSSTEPTVSSFDATYEVSGDNLEWAIGTVNEENANGSFEF
NCU00493.1	TVEYELTGGDTLKDVTVVIPYTSAEPQVSSFDAVYEVSGDSLEWSIGTVSEENASGSFEF
FG05436.1 	TVEYELTGGDALNDVSVVIPYAGSEPVISSYDATYDVSGDAIEWSIGNVDDENSTGSFEF
AN0922.1  	TVEYELTGSDSLRDVVVTIPYGTAEPAVSSFDAVYEVSGDSLDWNIGNVDENNASSSFEF
          	********.* *.** * ***  :** :**:**.*:**** ::* **.*.::*:..****

MG05676.1 	EAMADDENEFFPMQVRFSKTTPFVDVD0ISSVTLLEMNEEVTFSKDVRCTADTYLIE
NCU00493.1	EAQTDDENEFFPMQVRFSKSWPFVEVD0VQSVELVDEGEEVSFSKDIRSVADKFLIE
FG05436.1 	EAESGDENDFFPMTVRFNKSTPYIDVD0VNTASLLEEDEEVTFSKEVKSHADNFLVE
AN0922.1  	ESTADDENEFFPMTVRFSKASPFVEVD~VTNISLIEEGESTGYSKDIKSVAEGYLIE
          	*: :.***:**** ***.*: *:::** : .  *:: .*.. :**:::. *: :*:*
```
